# Extensive Chromosomal Reorganization in the Evolution of New World Muroid Rodents (Cricetidae, Sigmodontinae): Searching for Ancestral Phylogenetic Traits

**DOI:** 10.1371/journal.pone.0146179

**Published:** 2016-01-22

**Authors:** Adenilson Leão Pereira, Stella Miranda Malcher, Cleusa Yoshiko Nagamachi, Patricia Caroline Mary O’Brien, Malcolm Andrew Ferguson-Smith, Ana Cristina Mendes-Oliveira, Julio Cesar Pieczarka

**Affiliations:** 1 Laboratório de Citogenética, Centro de Estudos Avançados da Biodiversidade, ICB, Universidade Federal do Pará, Belém, Pará, Brasil; 2 CNPq Researcher, Brasília, Brasil; 3 Cambridge Resource Center for Comparative Genomics, Department of Veterinary Medicine, University of Cambridge, Cambridge, United Kingdom; 4 Laboratório de Zoologia e Ecologia de Vertebrados, ICB, Universidade Federal do Pará, Belém, Pará, Brasil; Universita degli Studi di Roma La Sapienza, ITALY

## Abstract

Sigmodontinae rodents show great diversity and complexity in morphology and ecology. This diversity is accompanied by extensive chromosome variation challenging attempts to reconstruct their ancestral genome. The species *Hylaeamys megacephalus*–HME (Oryzomyini, 2n = 54), *Necromys lasiurus—*NLA (Akodontini, 2n = 34) and *Akodon* sp.–ASP (Akodontini, 2n = 10) have extreme diploid numbers that make it difficult to understand the rearrangements that are responsible for such differences. In this study we analyzed these changes using whole chromosome probes of HME in cross-species painting of NLA and ASP to construct chromosome homology maps that reveal the rearrangements between species. We include data from the literature for other Sigmodontinae previously studied with probes from HME and *Mus musculus* (MMU) probes. We also use the HME probes on MMU chromosomes for the comparative analysis of NLA with other species already mapped by MMU probes. Our results show that NLA and ASP have highly rearranged karyotypes when compared to HME. Eleven HME syntenic blocks are shared among the species studied here. Four syntenies may be ancestral to Akodontini (HME2/18, 3/25, 18/25 and 4/11/16) and eight to Sigmodontinae (HME26, 1/12, 6/21, 7/9, 5/17, 11/16, 20/13 and 19/14/19). Using MMU data we identified six associations shared among rodents from seven subfamilies, where MMU3/18 and MMU8/13 are phylogenetic signatures of Sigmodontinae. We suggest that the associations MMU2entire, MMU6proximal/12entire, MMU3/18, MMU8/13, MMU1/17, MMU10/17, MMU12/17, MMU5/16, MMU5/6 and MMU7/19 are part of the ancestral Sigmodontinae genome.

## Introduction

Muroids are the most diverse group of extant rodents, with approximately 1500 species distributed in six families [1]. The families Cricetidae and Muridae are the most species rich [1–3].

The family Cricetidae is composed of six subfamilies [1]. Sigmodontinae comprises approximately 400 species with the tribes Akodontini, Abothrichini, Ichthyomyini, Oryzomyini, Phyllotini, Reithrodontini, Sigmodontini, Thomasomyini, Wiedomyini and Euneomyini, and 381 of these species are present in South America [1, 4–7]. Recent phylogenetic studies based on molecular data recognize this subfamily and its ten tribes as a monophyletic group [3, 7–10]. Two of these tribes are noteworthy for their taxonomic complexity, diversity and number of species. The most specie-rich tribe is Oryzomyini, with 118 species in 30 genera, and a distribution in rainforests to semi-arid regions of the Neotropical and Nearctic (southeastern section) regions [5, 11–13]. Akodontini is the second most speciose tribe, with 85 species in 15 genera, mainly in the tropical and sub-tropical forests of South America [1, 5].

G-banding is useful for the accurate identification of chromosomal homologies in karyotypes with few rearrangements, but is not useful in highly rearranged karyotypes, which makes it difficult to understand species with extensive chromosomal variation. Sigmodontinae have diploid numbers ranging from only 9–10 in species of genus *Akodon* to 92 in *Neusticomys ferreirai*, *Anotomys leander* and *Ichthyomys pittieri* [1, 14–16]. This large variation is problematic when trying to identify the chromosomal rearrangements between the extreme karyotypes in Sigmodontinae. However, chromosome painting has been very successful in demonstrating such rearrangements. This has been shown in *Akodon* species with diploid numbers varying from 10 to 44 by Ventura et al. [17], in *Akodon* and *Thaptomys* by Suarez et al [18] and by Swier et al [19] in *Sigmodon* genomes, which are quite stable, with few or no chromosome rearrangements. Nagamachi et al. [20] have used the same strategy to demonstrate that the Oryzomyini *Hylaeamys megacephalus* (2n = 54) and *Cerradomys langguthi* (2n = 46) are also highly rearranged. In addition, mouse whole chromosome probes were used to compare the karyotypes of the six Sigmodontinae species (five Akodontini and one Oryzomyini), and this enabled the reconstruction of chromosomal phylogeny and phylogenetic relationships [21–22]. However, not all segments had their homeology detected in some genomes (e.g.: *Necromys lasiurus*, *Thaptomys nigrita*, *Oligoryzomys flavescens*, *Akodon cursor*, *A*. *montensis*, *A*. *paranaensis* and *A*. *serrensis*; [21–22]). Recently Di-Nizo et al. [23] using whole chromosome probes of the *Oligoryzomys moojeni* (2n = 70), demonstrated that five species of the genus *Oligoryzomys* (Oryzomyini) have a high degree of chromosomal reorganization; not all existing homeologous were detected. The use of probes from different species, and the gaps left by these studies, make it difficult to comprehend all the mechanisms involved in the reconstruction of the ancestral Sigmodontinae karyotype (See [24]).

In this study, we constructed chromosomal homology maps between Akodontini *Akodon* sp. (2n = 10) and *Necromys lasiurus* (2n = 34) using cross species chromosome painting with Oryzomyini chromosomal probes from *Hylaeamys megacephalus* (2n = 54) to assess the mechanisms leading to the abrupt evolutionary rearrangements between species. We also compared our findings with those from the literature for species already mapped with *H*. *megacephalus* probes. Finally, we were able to compare our results on NLA using HME probes with some published results on NLA that used MMU probes. This allowed the identification of some corresponding regions of chromosome homology in studies made by different investigators using different probes. Our results reveal new findings for this important group of rodents and indicate new paths towards the reconstruction of the putative ancestral Sigmodontinae karyotype.

## Material and Methods

### Ethics Statement

JCP has a permanent field permit, number 13248 from “Instituto Chico Mendes de Conservação da Biodiversidade”. The Cytogenetics Laboratory from UFPa has a special permit number 19/2003 from the Ministry of Environment for the transport of samples and permit 52/2003 for using the samples in research. The Ethics Committee (Comitê de Ética Animal da Universidade Federal do Pará) approved this research. The specimens were captured using a live capture method designed for small mammals (traps type Sherman, Tomahawk and pitfalls [25]). Specimens were maintained in the lab with food and water, free from stress, until their euthanasia, made with the IP injection of barbiturates after local anesthetic (Ketamine HCl in combination with Diazepam).

### Specimen characteristics and Chromosome preparations

The specimens *Necromys lasiurus* (NLA, two males and one female) and *Akodon* sp. (ASP, one female and two males) were collected from the municipality of Parauapebas, Pará State, northern Brazil (Table 1). The sample was collected between October 2009 and January 2010. The identification of the specimens was made on the characteristics of skull and skin, and the voucher material deposited in the Mastozoology Collection of the Museu de Zoologia da Universidade Federal do Pará (MZUFPA). The chromosomal preparations were obtained from bone marrow after Colchicine treatment following Ford and Hamerton, [26]. We also obtained metaphases from a fibroblast cell culture of *Mus musculus* (MMU) in order to define some hybridizations not described previously in the literature.

**Table 1:** Species, diploid (2n), fundamental number (FN), sex, and collection localities of *Necromys lasiurus* and *Akodon* sp.

Voucher numbers	Species	2n	FN	Sex	Municipality/State	Geographic coordinate
UFPAM-160	*N*. *lasiurus*	34	34	M	Parauapebas/PA	02°57’08”S; 51°51’40”W
UFPAM-186	*N*. *lasiurus*	34	34	M	Parauapebas/PA	02°57’08”S; 51°51’40”W
UFPAM-201	*N*. *lasiurus*	34	34	F	Parauapebas/PA	02°57’08”S; 51°51’40”W
UFPAM-339	*Akodon* sp.	10	14	F	Parauapebas/PA	02°57’08”S; 51°51’40”W
UFPAM-143	*Akodon* sp.	10	14	M	Parauapebas/PA	02°57’08”S; 51°51’40”W
UFPAM-199	*Akodon* sp.	10	14	M	Parauapebas/PA	02°57’08”S; 51°51’40”W

Brazilian state: PA = Pará; M = Male and F = Female.

### Chromosomal banding

Conventional staining was used for diploid (2n) and fundamental number (FN) determination. G-banding followed the saline solution (2xSSC) incubation method [27]. The metaphases were stained with Wright’s solution after treatment with 2xSSC. C-banding was carried out according to Sumner [28].

### Fluorescence *in situ* Hybridization—FISH

The whole chromosome probes were derived from *Hylaeamys megacephalus* (HME; 2n = 54 and FN = 62) made by flow sorting from fibroblast cell culture chromosomes [20]. Of the 24 peaks, 21 correspond to a single chromosome pair, and 3 correspond to two chromosome pairs (HME(9,10), HME(13,22), HME(16,17); [19]). PCR products of sorted chromosomes from HME were labeled either with biotin-16-dUTP (Boehringer Mannheim), fluorescein isothiocyanate (FITC)-12-dUTP (Amersham) or Cy3-dUTP by taking 1μl of product to a second round of DOP-PCR using the same primer. The biotin probes were detected with avidin-Cy3 or avidin-FITC.

Chromosome painting was performed following the protocol previously described [20, 29], with some adaptations. Briefly, the slides were incubated in pepsin solution, and dehydrated in an ethanol series (70%, 90% and 100%), air-dried and aged in a 65°C incubator for two hours. Chromosomal DNA was denatured in 70% formamide/2xSSC at 70°C for 60 seconds, followed by preannealing the probes for 30 minutes at 37°C. The slides immersed immediately in cold 70% ethanol for 4 minutes followed by the ethanol series described above. After hybridization for 48–72 hours at 37°C (72–96 hours at 37°C for *Mus musculus*) and washing the slides (2x formamide 50%, 2x (2xSSC), 1x (4xSSC)/Tween at 38–40°C), the metaphases were stained with DAPI. Images were captured using the Axiovision 3.0 software with a CCD camera (Axiocam) coupled on a Zeiss-Axiophot 2 microscope or with a software Nis-Elements on a Nikon H550S microscope. Adobe Photoshop CS4 software was used for image processing.

### Analysis of shared syntenic blocks

We compare our results of cross-species painting to results from the species already mapped for *H*. *megacephalus* probes, namely *Cerradomys langguthi* (CLA; [20]), *Akodon montensis* (AMO) and *Tapthomys nigrita* (TNI, [18]), in order to demonstrate shared syntenic blocks in Sigmodontinae. The existing regions of homeology between the karyotypes of *Necromys lasiurus* and *Mus musculus* were taken from Hass et al. [22] and Guilly et al. [30]. This permitted a more complete comparative analysis between the karyotype of NLA and other species, which had been painted with MMU probes ([21–22, 24, 30–46]; S1 Table).

We extrapolate our results in *Akodon* sp. (ASP, 2n = 10) to the results of Ventura et al. [17] in *Akodon* sp. (ASP, 2n = 10) in which they used *Akodon paranaensis* (APA) probes. The homologies between the *Hylaeamys megacephalus* (HME) and *Akodon paranaensis* (APA) probes have been established by Suarez et al. [18] and are shown in S1 Table.

## Results

### Karyotypes and distribution of heterochromatin (HC)

*Necromys lasiurus* presented 2n = 34 and FN = 34, consisting of fifteen acrocentric and one small metacentric pair. The X and Y are a medium and small acrocentrics, respectively.

*Akondon* sp. presented 2n = 10 and FN = 14, consisting of two large metacentric pairs, a large acrocentric and a small metacentric pair. The X and Y are a medium and small acrocentrics, respectively.

The *Mus musculus* karyotype was standard with 2n = 20, in which all pairs are acrocentric.

In species NLA, ASP and MMU HC is located in the pericentromeric region of all chromosome pairs, the exception being the Y that is fully heterochromatic (data not shown).

### FISH with HME probes on NLA

**Fig 1:**
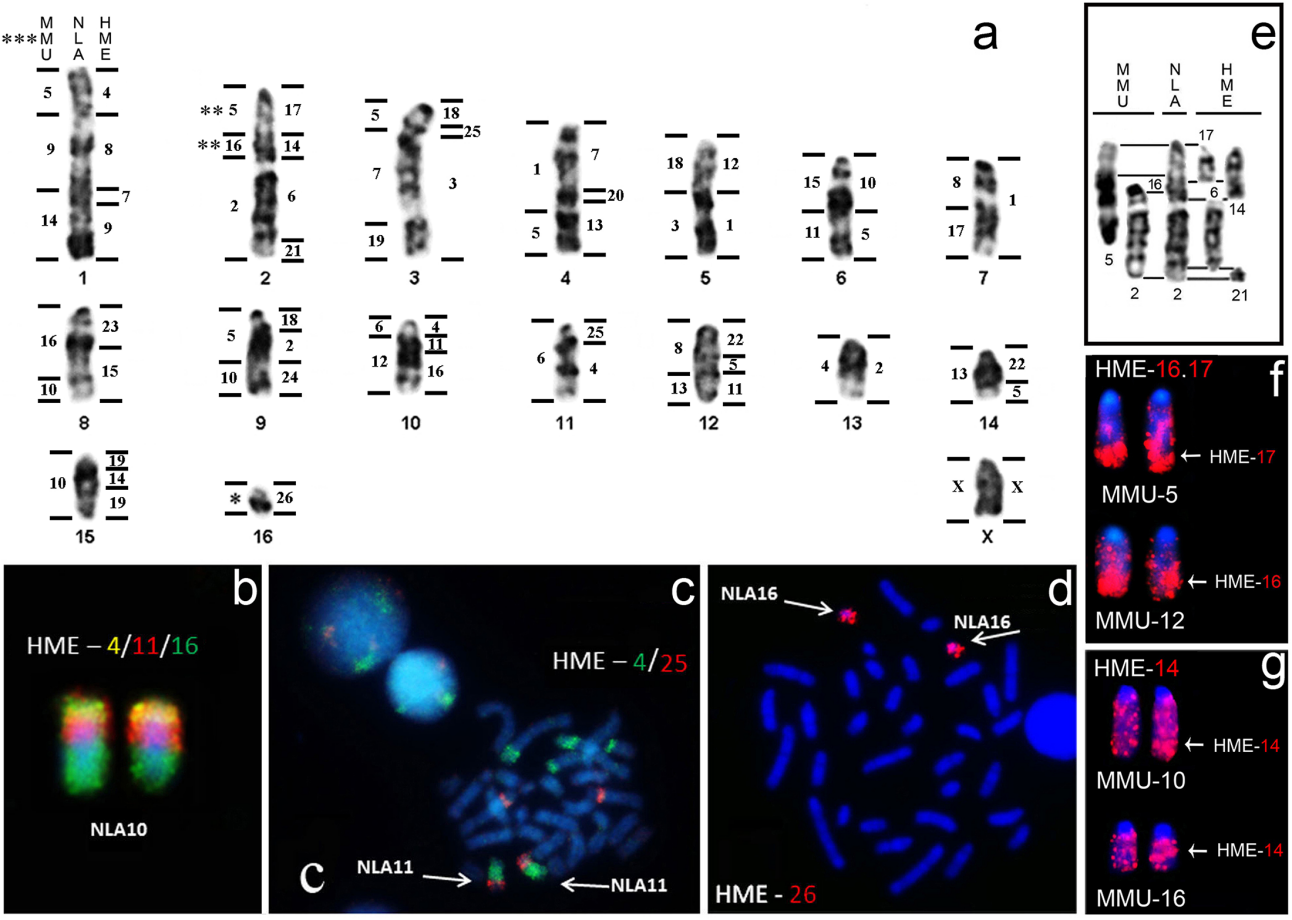
a) Comparative genomic mapping between NLA (2n = 34), MMU (2n = 40) and HME (2n = 54). Examples of FISH: b) Association HME4/11/16; c) Association HME4/25; d) HME26; e) A comparison of G-banding conserved regions between NLA2, HME6, HME14, HME17, HME21, MMU2, and MMU5 (MMU16 is mentioned because of the homeology, but did not remains G-banding conserved in NLA2proximal); f) MHE(16,17) hybridized onto MMU5 and MMU12; g) HME14 hybridized onto MMU10 and MMU16. (*) Region that did not hybridize with any MMU probe. (**) Regions homologues to MMU genome identified in this study (Association MMU5/16). (***) Mapping between MMU and NLA adapted from Hass et al. [22].

All probes from HME (2n = 54 and FN = 62) hybridized to metaphases of NLA (2n = 34 and FN = 34), and revealed 40 homologous segments in the genome of this species (Fig 1a–1d and S1 Fig). Almost all chromosome pairs of NLA hybridized with more than one HME probe, the exceptions being NLA7, 13, 16 and X that hybridized with HME1, 2, 26 and X (Fig 1a), respectively. Sixteen probes (HME3, 6, 8, 9, 10, 12, 13, 15, 16, 17, 20, 21, 23, 24, 26 and X), demonstrated only one homologous region in the NLA karyotype, while probes HME7, 11, 14, 18, 19, 22 and 25, each presented two hybridization signals in NLA (Fig 1a). HME4 and HME5 presented three hybridization signals (Fig 1a). A total of 13 HME shared associations were detected in NLA: HME4/8/7/9 (NLA1), HME17/14/6/21 (NLA2), HME18/25/3 (NLA3), HME7/20/13 (NLA4), HME1/12 (NLA5), HME 5/10 (NLA6), HME15/23 (NLA8), HME18/2/24 (NLA9), HME4/11/16 (NLA10), HME4/25 (NLA11), HME11/5/22 (NLA12), HME5/22 (NLA14) and HME19/14/19 (NLA15) (Fig 1a–1c).

### FISH with HME probes on ASP

**Fig 2:**
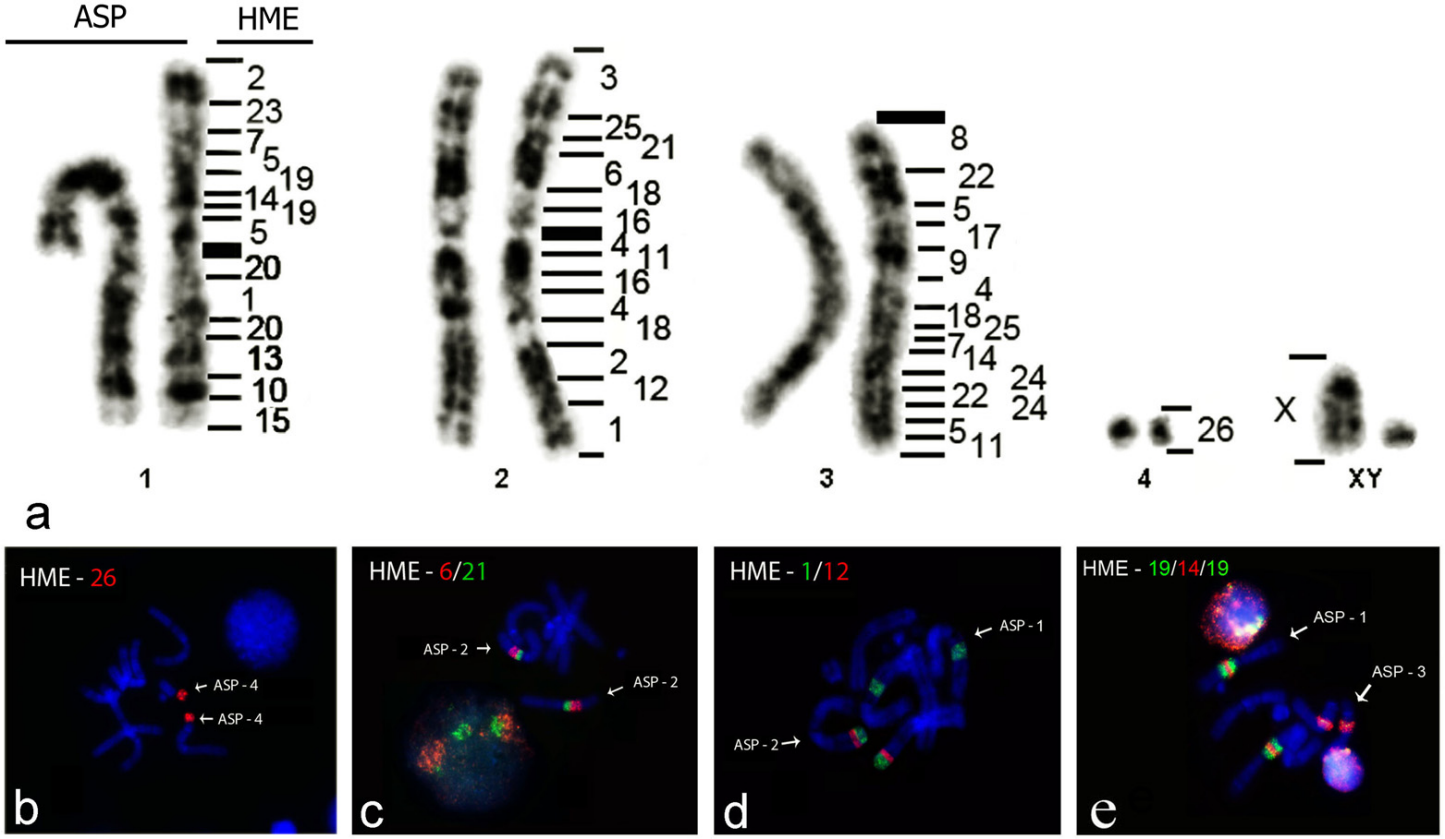
a) Comparative genomic mapping between *Hylaeamys megacephalus* (HME; 2n = 54) and *Akodon* sp. (ASP; 2n = 10). Examples of FISH: b) HME26 in ASP4; c) HME6/21 association in ASP2; d) HME1/12 association in ASP2 and e) HME19/14/19 association in ASP1.

All probes from HME hybridized to metaphases of ASP (2n = 10 and FN = 14), revealing 45 homologous segments (Fig 2a–2e and S2 Fig). Almost all chromosome pairs of ASP hybridized with more than one HME probe, the exceptions being ASP4 and X, corresponding to HME26 and HMEX, respectively (Fig 2a). Thirteen chromosomes (HME3, 6, 8, 9, 10, 12, 13, 15, 17, 21, 23, 26 and X), were homologous to only one region each, while the chromosomes HME1, 2, 4, 7, 11, 14, 16, 19, 20, 22, 24 and 25, each presented two hybridization signals (Fig 2a–2e). HME(16,17), 18 and (13,22) gave three hybridization signals, while probe HME5 hybridized to four regions (Fig 2a). Three complex associations were found: HME2/23/7/5/19/14/19/5/20/1/20/13/10/15 (ASP1), HME3/25/21/6/18/16/4/11/16/4/18/2/12/1 (ASP2) and HME8/22/5/17/9 /4/18/25/7/14/24/22/24/5/11 (ASP3)) (Fig 2a).

### FISH of probes HME14, HME(16,17) and HME26 on MMU

Since the homologies between NLA2proximal+medial, NLA16 and the genome of MMU were not defined previously [22], we hybridized HME14, HME(16,17) and HME26 to MMU chromosomes to demonstrate that these regions are homologous to NLA2 and NLA16 (Fig 1a).

The probe HME14 showed two signals, in MMU10 and MMU16 (Fig 1g); HME(16,17) also showed two signals, with HME16 homeologous to MMU12 and HME17 to MMU5 by FISH and G-banding (Fig 1f and S3 Fig); HME26 did not show any signal in MMU.

### Shared syntenic blocks among NLA, ASP, AMO, TNI and CLA

The comparisons between NLA, ASP, CLA, AMO and TNI allowed the identification of 11 syntenic blocks of HME shared by these rodents (Table 2). Three blocks are shared by all the species analyzed (HME26, HME6/21 and HME20/13; Table 2). The association HME1/12 is absent in TNI but is present in the other species (Table 2). Only CLA does not share the association HME3/25 with the other species in this study (Table 2). The association HME2/18 is shared by NLA, ASP and AMO (Table 2). HME18/25 is shared by NLA, ASP and TNI (Table 2). The association HME7/9 is shared by CLA, NLA (HME7/9 in NLA) and AMO (Table 2). The association HME4/11/16 is shared by NLA (HME4/11/16 in NLA), ASP and AMO, while the association HME11/16 is found only in CLA and TNI (Table 2). The association HME5/19/14/19/5 is shared by CLA and ASP, while NLA and AMO has only the segment HME19/14/19 and TNI has HME 14/19 (Table 2). The association HME5/17/22 is found in CLA, while in ASP it is HME17/5/22 (Table 2). In NLA there is only HME5/22 (HME5/22 in NLA12 and NLA14; Fig 1a and Table 2).

**Table 2:** *Hylaeamys megacephalus* (HME) syntenic blocks in other Sigmodontinae.

Family Cricetidae: Subfamily Sigmodontinae					
	Tribe Oryzomyini	Tribe Akodontini			
Character	CLA^a^	NLA^b^	ASP^b^	AMO^c^	TNI^c^
HME26	+	+	+	+	+
HME1/12	+	+	+	+	-
HME2/18	-	+	+	+	-
HME3/25	-	+	+	+	+
HME6/21	+	+	+	+	+
HME18/25	-	+	+	-	+
HME7/9	+	+	-	+	-
HME20/13	+	+	+	+	+
HME4/11/16	HME11/16	+	+	+	HME11/16
HME5/19/14/19/5	+	HME19/14/19	+	HME19/14/19	HME14/19
HME5/17/22	+	HME5/22	HME22/5/17	HME22/5/17	-

CLA = *Cerradomys langguth*; ASP = *Akodon* sp.; NLA = *Necromys lasiurus* (NLA); *Akodon montensis* (AMO); *Tapthomys nigrita* (TNI).

(+) = presence; (–) = lack of character.

^(a)^ Data from Nagamachi et al. [20].

^(b)^ This study.

^(c)^ Data from Suarez et al. [18].

### Syntenic blocks shared among NLA, HME and MMU

After identification of the homologous segments in the karyotypes of these species through chromosome painting, comparison by G-bands (GB) was performed (S3 Fig). Thirteen chromosomes of NLA (NLA1, 2, 3, 4, 5, 6, 7, 8, 10, 11, 12, 13 and 16) were conserved by GB across the homeologous segments in HME and MMU (S3 Fig). The comparative analysis by GB allowed us to identify HME(9,10), HME(13,22) and HME(16,17) with their corresponding segments in NLA (S3 Fig and Fig 1a). For instance, HME(9,10) is homologous to NLA1distal (HME9) and NLA6proximal (HME10; Fig 1a and S3 Fig); HME(13,22) to NLA4distal (HME13; S3 Fig) and to NLA12proximal and NLA14proximal (HME22; Fig 1a); HME(16,17) to NLA2proximal (HME17; Fig 1a and S3 Fig) and NLA10medial+distal (HME16; Fig 1a and S3 Fig). The NLA2proximal is homologous to MMU5 and MMU16 (Fig 1a–1g and S3 Fig), while NLA2medial+distal was found to be homologous to MMU2 (Fig 1e–1g and S3 Fig). We identified two regions of the genome of MMU (MMU1distal and MMU11proximal), which were not identified by Hass et al. [22] during the mapping between NLA and MMU (Fig 1a and S3 Fig). Three chromosomes (NLA 9, 14 and 15) and some chromosome regions in NLA did not present a conserved GB pattern when compared to the genomes of HME and MMU (S3 Fig), though FISH clearly demonstrated the homology between them (Fig 1a).

### Shared syntenic blocks among subfamilies

We compared NLA to 35 species belonging to different subfamilies of rodents in the superfamily Muroidea that were previously studied with MMU probes ([21–22, 24, 30–46]; S2 Table). A total of six shared syntenic blocks were found: MMU5/9, MMU5/7/19, MMU5/10, MMU3/18, MMU8/13 and MMU6/12 (S2 Table). The association MMU5/9 is shared among three Cricetidae (*N*. *lasiurus*, *Cricetulus griseus* and *Cricetus cricetus*; [22, 40, 45–46]), two Muridae (*Nannomys mattheyi* [44] and *Acomys dimidiatus* [38]; S2 Table). The association MMU5/10 is shared with NLA and four Arvicolinae species (*Ellobius lutescens*, *Ellobius talpinus*, *Microtus agrestis* and *Microtus oeconomus*; [41–42]; S2 Table). In this subfamily MMU5/10 is associated with MMU7 (MMU7/5/10; [41–42]; S2 Table). MMU7/19 is shared by all species analyzed here ([21–22, 24, 30–46]; S2 Table). MMU3/18 e MMU8/13 can be found in almost all Sigmodontinae, but is not found in any other species we studied ([21–22]; S2 Table). The association MMU6/12 is found in five species of Sigmodontinae (*Akodon cursor*, *Akodon montensis*, *Akodon paranaenses*, *Akodon serrensis*, *Necromys lasiurus* and *Thaptomys nigrita*; [21–22]; S2 Table) and four Arvicolinae (*Ellobius lutescens*, *Ellobius talpinus*, *Microtus agrestis* and *Microtus oeconomus*; [41–42]; S2 Table). However, our joint analysis with FISH and GB demonstrated that this association is different in each subfamily, being MMU6proximal/12entire in Sigmodontinae and MMU6medial+distal/12proximal in Arvicolinae (S2 Table).

## Discussion

### The genome of NLA

The karyotype of NLA (2n = 34/FN = 34) mapped here with HME probes is the same as previously mapped with MMU probes [22]. In that study not all MMU probes hybridized to NLA, because the long time after the separation of the two species, between 23.3 and 24.7 Mya [3, 22], probably resulted in a high divergence of DNA sequences.

In the present study we found all syntenies between the karyotypes of HME and NLA, including rearrangements not found before in mapping between MMU and NLA [22], such as the insertion or inversion that led to NLA15 (HME19/14/19; Fig 1a) or the translocation that led to NLA14 (HME5/22; Fig 1a). In the map of Hass et al. [22], chromosome NLA16 did not show any homology with MMU. Here NLA16 was homologous to HME26, showing conserved synteny by both FISH and GB (Fig 1a and S3 Fig). Furthermore, we find that MMU2 is homologous only to NLA2medial+distal, and not to all NLA2 as suggested by Hass et al. [22] (Fig 1e); the MMU2proximal is homologous to MMU5 and MMU16 (Fig 1a and 1e–1g). Therefore, we redefined the existing homology between MMU and NLA2, where NLA2 is homologous with the association MMU5/16/2 (Fig 1a and 1e–1g).

Our comparative analyses for FISH and G-banding among NLA, HME and MMU revealed that NLA1 is the result of an *in tandem* fusion between MMU5 and MMU9 and a Robertsonian translocation between MMU9 and MMU14 (S3 Fig). NLA2 results from three *in tandem* fusions of HME17, HME14 and HME6 and a Robertsonian translocation between HME6 and HME21 (S3 Fig). Part of NLA3 results from an *in tandem* fusion between MMU7 and MMU19 (S3 Fig). NLA4 results from three *in tandem* fusions of HME7, HME20 and HME13 (S3 Fig). NLA5 results from the *in tandem* fusions of MMU18 and MMU3 and of HME12 and HME1 (S3 Fig). The other NLA pairs are the result of other rearrangements like translocations, insertions and inversions (Fig 1a and S3 Fig). These *in tandem* fusions and translocations support the Barros et al. [47] hypothesis that NLA reduced its diploid number during the Akodontini radiation.

### The genome of ASP

The genomes of HME and ASP are highly reorganized (Fig 2a–2e). The low diploid number in ASP results from many fusions, translocations and insertions mainly in pairs ASP1, ASP2 and ASP3 (Fig 2a). In ASP1 it was found that the association HME19/14/19, resulted from an inversion or insertion. This same segment is part of another inversion or insertion and includes HME5, being HME5/19/14/19/5 (Fig 2a and 2e). Apart from this, HME1 also has an insertion or is involved in an inversion of HME20, becoming HME20/1/20 in ASP1 (Fig 2a). In ASP2, there is an insertion or inversion of HME11/16 in HME4 resulting in HME4/11/16/4 (Fig 2a). The association HME16/4/11/16/4 has an insertion or inversion of HME18 becoming HME 18/16/4/11/16/4/18 in ASP2 (Fig 2a). Meanwhile, ASP3 had only one inversion or insertion of HME22 in HME24, becoming HME24/22/24 (Fig 2a). The other rearrangements were fusions and/or translocations in ASP1, ASP2 and ASP3, causing the diploid number reduction in ASP (Fig 2a).

**Fig 3:**
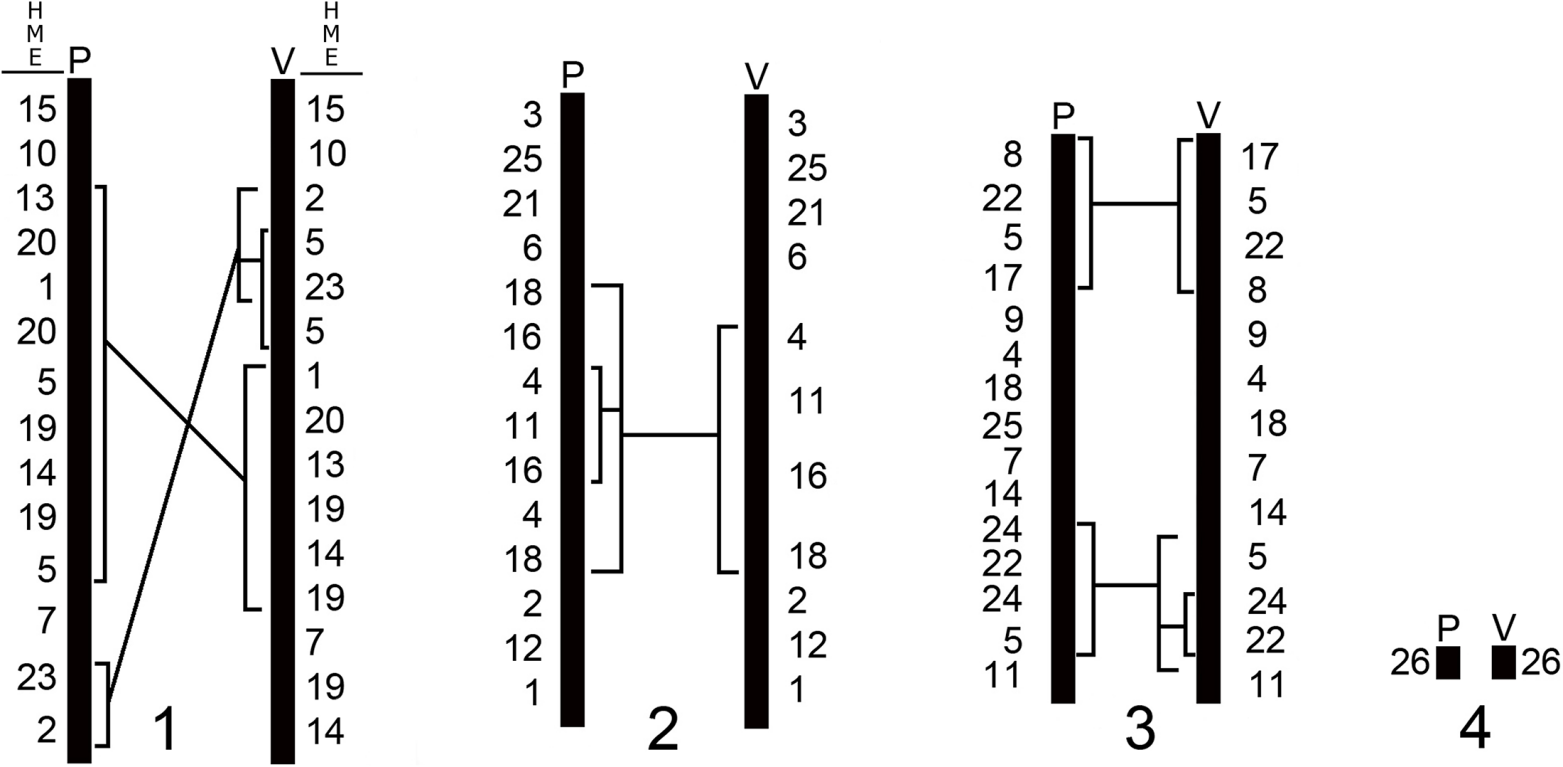
a) Comparative mapping between *Akodon* sp. (ASP; 2n = 10) described here and by Ventura et al. [17]. The existing homology between the karyotype ASP (2n = 10) studied by Ventura et al. [17] and HME was determined and based upon extrapolation of our data to data in the literature, as established in S1 Table. HME = *Hylaeamys megacephalus*; P = Pereira et al. [present study]; V = Ventura et al. [17].

The karyotype of *Akodon* sp. (2n = 10) studied here has the same diploid number, a similar morphology and the same syntenic groups of *Akodon* sp. (2n = 10) as were reported by Ventura et al. [17]. The extrapolation of our mapping in ASP to the mapping performed by Ventura et al. [17] in ASP (S1 Table) revealed that the two karyotypes have syntenic groups distributed in a different order (Fig 3). We observe that the chromosome pairs are homologous, but the chromosome ASP1 differs in three complex rearrangements, such as inversions and/or insertions involving large syntenic blocks (Fig 3). ASP2 differs by two inversions and/or insertions surrounding the block HME18/16/4/11/16/4/18 (ASP2, this study; Fig 3) or HME4/11/16/18 (ASP2, [17]; Fig 3). ASP3 differs by an inversion involving HME8/22/5/17 (Fig 3) and an inversion and/or insertion occurring in block HME24/22/24/5 (ASP3; this study) or HME5/24/22 ([17]; Fig 3). ASP3 differs also for a possible translocation, since in our sample there is a segment of HME25, not found in the sample of Ventura et al. [17]. The fourth pair and the sex chromosomes do not show any differences. These rearrangements suggest that the animals in the two reports are from different species, despite their similar karyotypes. The samples were collected from localities that are one thousand kilometers apart. It is noteworthy that the diploid number is the same and that most of the rearrangements are intrachromosomal. It may be that there is a mechanism that maintains a stable diploid number. These rearrangements may contribute to reproductive isolation, since they can cause meiotic problems during gametogenesis in eventual hybrids generated from these two cytotypes [48–50].

### The genome of NLA *vs*. ASP

**Fig 4:**
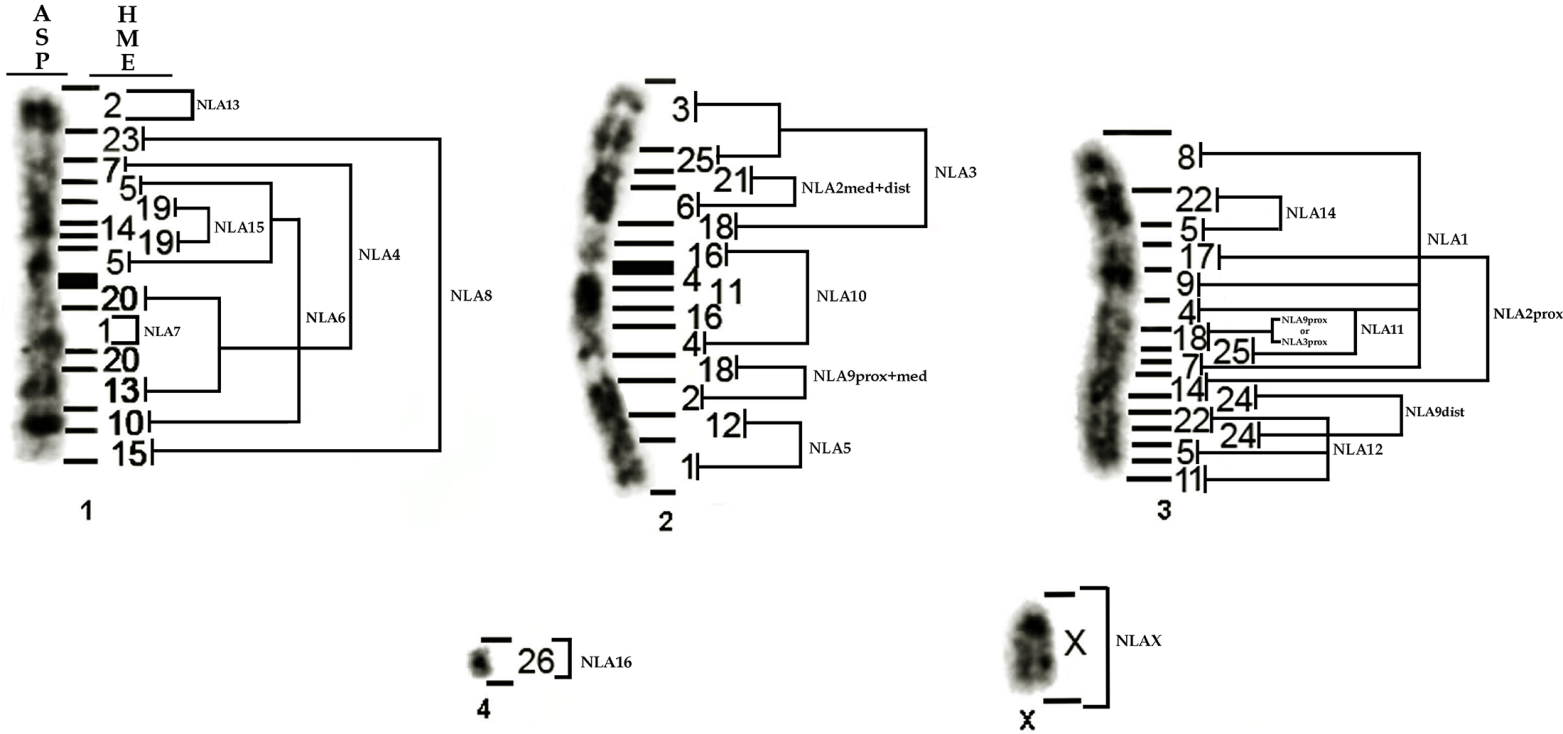
Karyotype of *Akodon* sp. (ASP, 2n = 10) and its homologies to *Necromys lasiurus* (NLA, 2n = 34). HME = *Hylaeamys megacephalus*. prox = Proximal, med = Medial, dist = Distal.

Our comparison between NLA and ASP revealed that the three major pairs of ASP (ASP1, ASP2 and ASP3) originated from complex rearrangements involving multiple insertions, translocations, fusions *in tandem* and inversions involving NLA pairs (Fig 4).

ASP1 results from multiple insertions or inversions, fusions and translocations involving NLA4, NLA6, NLA7, NLA8, NLA13 and NLA15 (Fig 4). ASP2 results from an insertion or inversion of NLA2medial+distal in NLA3, followed by fusions *in tandem* of NLA10, NLA9proximal+medial and NLA5 (Fig 4). The syntenic block corresponding to NLA10 (HME4/11/16 in NLA; Fig 1a) in ASP2 has one or two inversions leading to HME16/4/11/16/4 (NLA10 in ASP2; Fig 4). The third pair (ASP3) has at least two insertions (NLA14 and NLA9proximal or NLA3proximal) and many inversions involving NLA1, NLA9distal, NLA2proximal, NLA11, NLA12 and NLA14 (Fig 4). The fourth pair (ASP4) and the X remained conserved between the two species (Fig 4).

Our data suggest that ASP had its diploid number reduced when compared to NLA, in agreement with Ventura et al. [17]. Barros et al. [47] suggested that the ancestral karyotype in Akodontini had a high diploid number (2n = 52) and had a tendency to reduction in many species, as in ASP and NLA. When compared to the Oryzomyini HME (2n = 54) our data are in agreement with this proposition.

### The genome of Sigmodontinae

The 11 syntenic blocks in HME that are shared with other Sigmodontinae can be potential markers in phylogenetic analyses (Table 2). The associations HME2/18, HME3/25 and HME18/25 seem to be shared only by Akodontini, suggesting that these characters are unique to that tribe (Table 2). HME 26, HME1/12, HME6/21 and HME20/13 are shared for almost all species studied here (TNI does not share HME1/12; Table 2) and possibly are part of the ancestral genome of Sigmodontinae. HME7/9 is found in CLA (Oryzomyini), is shared with NLA (HME7/9 in NLA) and AMO (Akodontini) and probably is part of the ancestral genome of Sigmodontinae too (Table 2). HME4/11/16 is found in NLA (HME4/11/16 in NLA; Table 2), ASP and AMO, while in CLA and TNI only HME11/16, is found (Table 2). This difference can be the result of a translocation of HME4 in CLA and TNI to another region of its genome, or the translocation of HME4 to HME11/16 in NLA, ASP and AMO. Probably HME11/16 is the ancestral form, since it is found both on Oryzomyini and Akodontini, while HME4/11/16 can be an Akodontini trait (Table 2). The association HME5/19/14/19/5 is shared by CLA and ASP, while NLA and AMO has only the segment HME19/14/19 (Table 2), maybe resulting from an insertion or inversion of HME19/14/19 in HME5 in CLA and ASP. TNI has HME14/19, which may have originated from an inversion or translocation in HME19/14/19 (Table 2). We believe that HME19/14/19 is an ancestral character of Sigmodontinae as it is present in Oryzomyini and Akodontini (Table 2). The most variable segment is HME5/17/22 in CLA, and HME17/5/22 in ASP (Table 2). This difference may be the result of an inversion in CLA or ASP. In NLA there is HME5/22 probably because of a translocation of HME17 to another region. It is not possible to determine the ancestral position of this block.

Here, we define for the first time a set of syntenic blocks that probably were part of the putative ancestral Sigmodontinae karyotype. Further analysis of a greater number of species belonging to different tribes will complement this reconstruction. For the present we suggest that the blocks HME2/18, HME3/25, HME18/25, HME4/11/16, are ancestral to Akodontini. The blocks HME26, HME1/12, HME6/21, HME20/13, HME7/9, HME5/17, HME11/16 and HME19/14/19 are probably ancestral to Sigmodontinae.

### Syntenic blocks of MMU ancestral to Sigmodontinae

The associations MMU5/9 and MMU5/10 are not exclusive to NLA as suggested by Hass et al. [22], since MMU5/9 is found in *Cricetulus griseus* and *Cricetus cricetus* (Cricetidae: Cricetinae [40, 46]), in *Nannomys mattheyi* (Muridae: Murinae; [44]) and *Acomys dimidiatus* (Muridae: Deomyinae; [38]); MMU5/10 in NLA is also shared by *Ellobius lutescens*, *Ellobius talpinus*, *Microtus agrestis* and *Microtus oeconomus* (Cricetidae: Arvicolinae, [41–42]; S2 Table). In addition, MMU5/7 is not shared only by *N*. *lasiurus* and *Akodon cursor* as suggested by Hass et al. [22], but also by Arvicolinae species (*Ellobius lutescens*, *Ellobius talpinus*, *Microtus agrestis* and *Microtus oeconomus*, [41–42]; S2 Table). These traits could arise in an independent fashion by homoplasy, or result from a polymorphic trait that is fixed in different taxa by hemiplasy [51].

MMU6/12 is found in Sigmodontinae and Arvicolinae. However, MMU6proximal/12entire is found only in Sigmodontinae species (*Akodon cursor*, *Akodon montensis*, *Akodon paranaensis*, *Akodon serrensis*, *Necromys lasiurus* and *Thaptomys nigrita*, [21–22]; S2 Table), while MMU6medial+distal/12proximal is found in Arvicolinae species (*Ellobius lutescens*, *Ellobius talpinus*, *Microtus agrestis* and *Microtus oeconomus*, [41–42]; S2 Table). These blocks are potentially phylogenetic signatures for these two subfamilies.

MMU7/19 is highly conserved in Muroidea, as shown by all species analyzed here ([21–22, 24, 30–46]; S2 Table), and probably is found in the ancestral genome of this group [24, 32, 40, 46, 52]; S2 Table). This association is present in the human genome (HSA11 and HSA10) and in the Bovidae genome (BTA26) [43, 53]. These data suggest that this block exists in the Eutherian ancestral karyotype.

MMU3/18 is shared by all Akodontini (*Akodon cursor*, *Akodon montensis*, *Akodon paranaensis*, *Akodon serrensis*, *Necromys lasiurus* and *Thaptomys nigrita*; [21–22] and is absent in the only Oryzomyini species (*Oligoryzomys flavescens*) analyzed by Hass et al. [21] (S2 Table). This suggests that this trait could be exclusive to Akodontini. However, our analysis shows that this association is present in NLA5 (Fig 1a), corresponding to HME1/12. In addition HME1/12 was found also in ASP2 (this study), AMO1 (Akodontini; [18]) and CLA2 (Oryzomyini; [19]) (S2 Table), showing that it is not exclusive to Akodontini. Thus, we consider that MMU6proximal+12entire (only in Akodontini tribe), MMU3/18 and MMU8/13 are chromosomes signatures exclusive to the subfamily Sigmodontinae.

Many MMU associations found in Sigmodontinae, like MMU5/6, MMU1/17, MMU10/17, MMU12/17 in *Akodon cursor* [21], MMU5/6 in *Oligozyzomys flavescens* [21], MMU16/17 in *Thaptomys nigrita* [22] and MMU5/16 in *Necromys lasiurus* (this study), are also found in the ancestral karyotypes of Cricetinae, Arvicolinae, Murinae and Muroidea [24, 32, 46]. Furthermore, MMU2entire (HME6/21) present in *Akodon cursor*, *Akodon montensis*, *Akodon paranaensis*, *Akodon serrensis*, *Oligoryzomys flavescens*, *Necromys lasiurus* [21–22] and *Akodon* sp. (this study) is also found in the ancestral karyotype shared by Cricetinae and Arvicolinae and the ancestral karyotype of Muroidea [24, 32, 46]. These blocks probably are part of the ancestral karyotype of Sigmodontinae.

The huge variability of the genome structure of Sigmodontinae makes the reconstruction of the ancestral karyotype by classic cytogenetics (and so the understanding of the evolutionary history of its genome) an impossible task. However, the chromosome painting technique allows the precise visualization of the homology of syntenic blocks in many species. At the moment there are only a few studies in Sigmodontinae that use this approach, but these studies already have provided some relevant information. We have used the MMU genome as a reference for rodents and can now suggest that MMU2entire, MMU6proximal/12entire, MMU3/18, MMU8/13, MMU1/17, MMU10/17, MMU12/17, MMU5/16, MMU5/6 and MMU7/19 are part of that ancestral Sigmodontinae genome.

## Supporting Information

S1 Fig Hybridization of each *Hylaeamys megacephalus* whole chromosome probe on chromosome pairs of *Necromys lasiurus*. (JPG)

S2 Fig Hybridization of each *Hylaeamys megacephalus* whole chromosome probe on chromosome pairs of *Akodon* sp. (JPG)

S3 Fig a) G-banded conserved regions between NLA, HME and MMU. (*) Asterix indicate the regions MMU1distal and MMU11proximal not found in the mapping of Hass et al. [21] in NLA. Arrow indicates the region, which does not hybridize to any MMU. Invers: inversion. b) Chromosomes of NLA (NLA9, NLA14 and NLA15) which homologous segments were identified by FISH but are not G-banding conserved. Dark lines delimit the conserved regions. Adapted from Nagamachi et al. [19] and Guilly et al. [29].
(JPG)

S1 Table Homeologies among *Akodon paranaensis* (APA; [17]) and *Hylaeamys megacephalus* (HME; [19]) whole chromosome probes according to Fig 3A in Suarez et al. [18]. (DOC)

S2 Table Analysis of syntenic blocks of *Mus musculus* shared between rodents Muroideos the New and Old World based on literature data. Key to abbreviations: MMU = *M*. *musculus*; prox = proximal; med = medium and dist = distal.
(DOC)
