# Supplementary figures and images for: Extensive Chromosomal Reorganization in the Evolution of New World Muroid Rodents (Cricetidae, Sigmodontinae): Searching for Ancestral Phylogenetic Traits
Source: PLoS One. 2016 Jan 22;11(1):e0146179. doi: 10.1371/journal.pone.0146179 (PMC4723050; doi:10.1371/journal.pone.0146179)

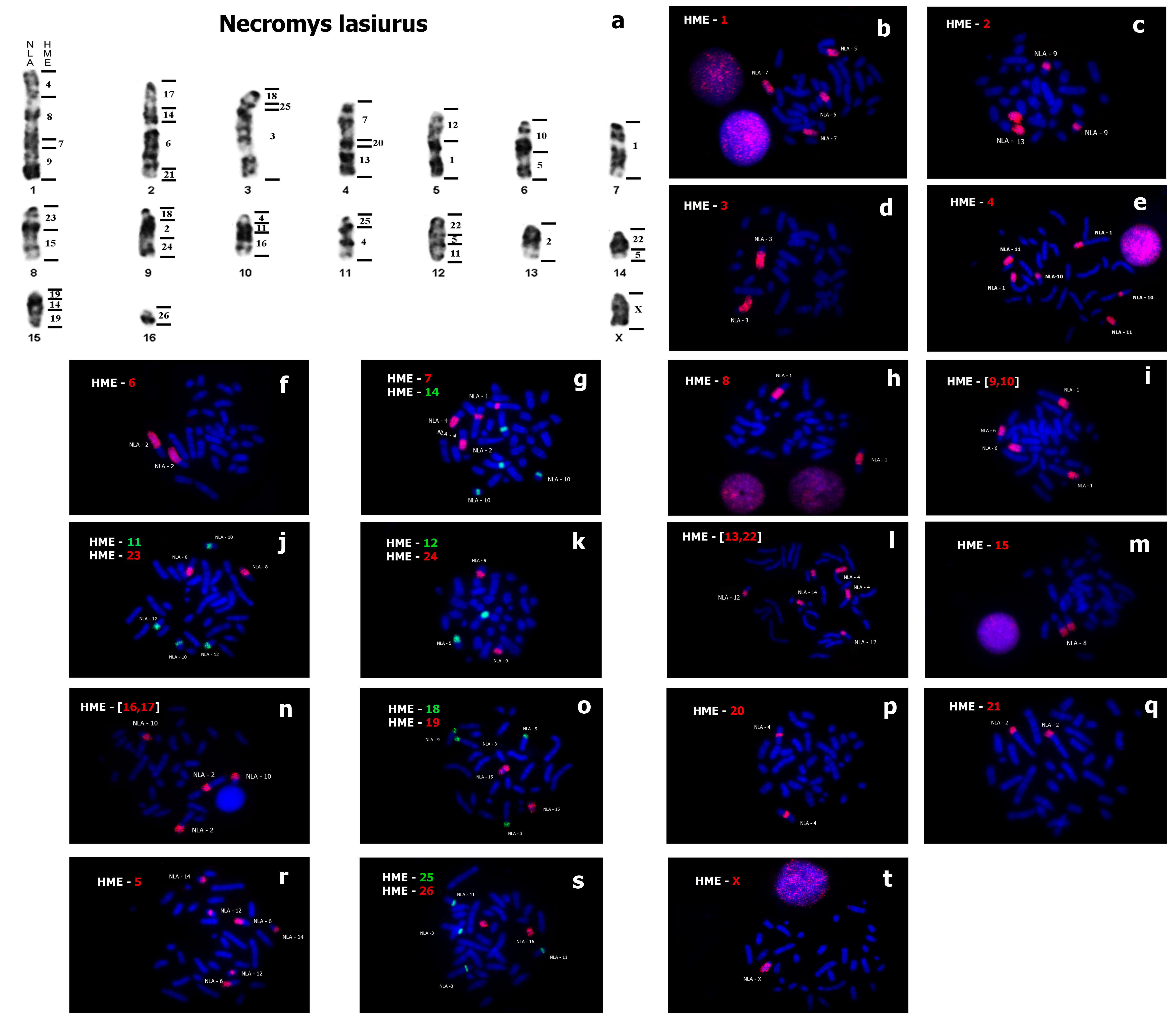

Supplement: S1 Fig — (JPG) [file pone.0146179.s001.jpg]

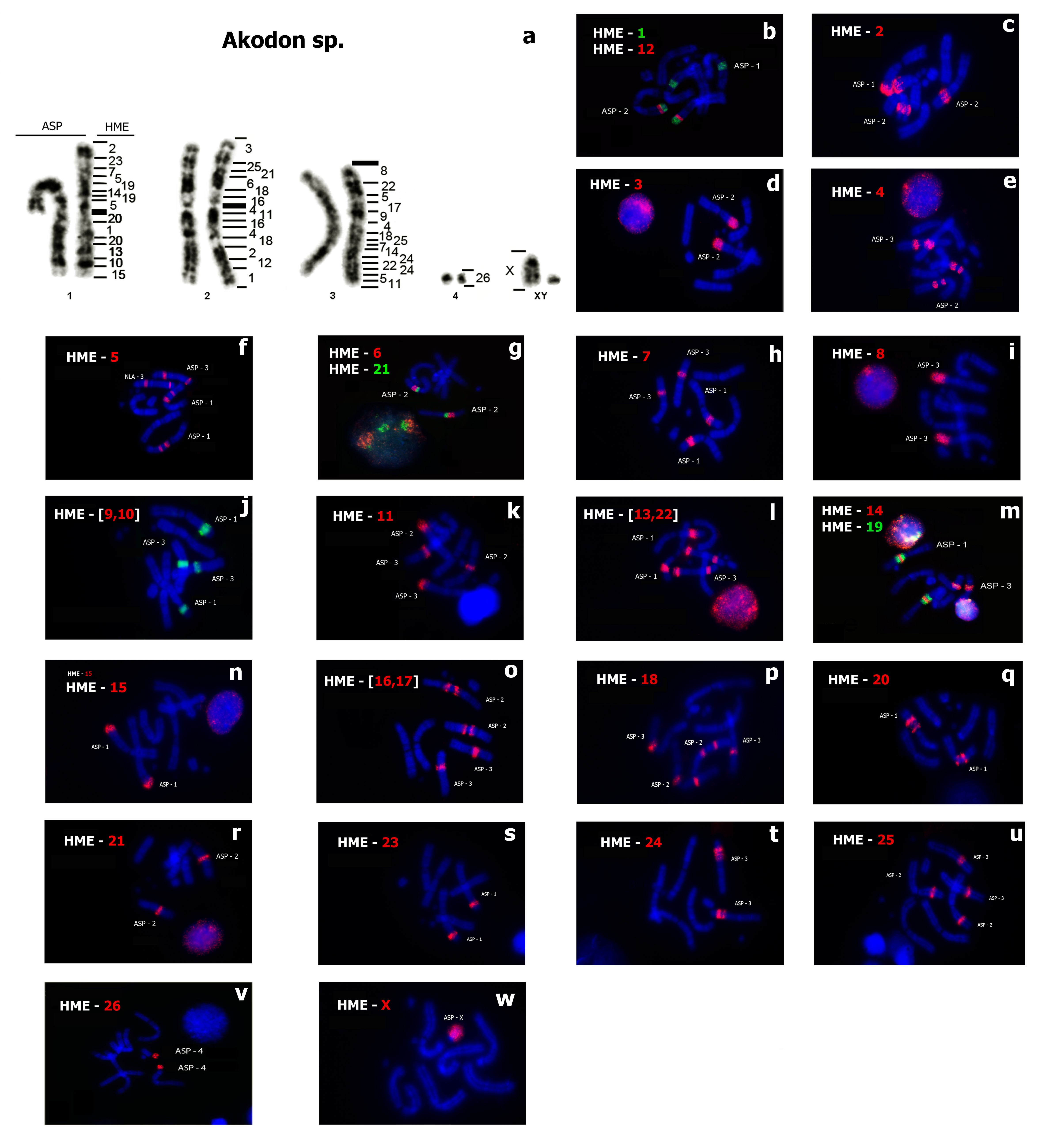

Supplement: S2 Fig — (JPG) [file pone.0146179.s002.jpg]
