# Supplementary material for: Extensive Chromosomal Reorganization in the Evolution of New World Muroid Rodents (Cricetidae, Sigmodontinae): Searching for Ancestral Phylogenetic Traits
Source: PLoS One. 2016 Jan 22;11(1):e0146179. doi: 10.1371/journal.pone.0146179 (PMC4723050; doi:10.1371/journal.pone.0146179)
Supplement: S1 Table — (DOC) [file pone.0146179.s004.doc]

Table S1: Homeologies among *Akodon paranaensis* (APA; [17]) and *Hylaeamys megacephalus* (HME; [20]) whole chromosome probes according to Figure 3A in Suarez et al. [18].

| **APA** |  | **HME** |  | **APA** |  | **HME** |  | **APA** |  | **HME** |
| --- | --- | --- | --- | --- | --- | --- | --- | --- | --- | --- |
| 1 |  | 1, 2, 12 |  | 8 |  | 7, 9,10 |  | 15 |  | 7, 19 |
| 2 |  | 3, 25 |  | 9 |  | 4,18 |  | 16 |  | 23 |
| 3 |  | 4, 11, (16,17) |  | 10 |  | (13,22), 20 |  | 17 |  | 14, 19 |
| 4 |  | 6, 21 |  | 11 |  | 11, (13,22) |  | 18 |  | 9,1 |
| 5 |  | 1 |  | 12 |  | 5, 14, 24 |  | 19 |  | 5 |
| 6 |  | 5, (13,22), (16,17) |  | 13 |  | 15 |  | 20 |  | 18 |
| 7 |  | 8 |  | 14 |  | 2 |  | 21 |  | 26 |
