# Supplementary material for: Extensive Chromosomal Reorganization in the Evolution of New World Muroid Rodents (Cricetidae, Sigmodontinae): Searching for Ancestral Phylogenetic Traits
Source: PLoS One. 2016 Jan 22;11(1):e0146179. doi: 10.1371/journal.pone.0146179 (PMC4723050; doi:10.1371/journal.pone.0146179)
Supplement: S2 Table — Key to abbreviations: MMU = M. musculus; prox = proximal; med = medium and dist = distal. (DOC) [file pone.0146179.s005.doc]

Supporting information: S2 Table

**Table S2: Analysis of syntenic blocks of *Mus musculus* shared between Muroid rodents the New and Old World based on literature data. Key to abbreviations: MMU= *M. musculus*; prox= proximal; med= medium and dist= distal.**

| **Taxon** | **2n** | **Character** | | | | | | | | | | | | | | | | **Reference**** | | | | | |
| --- | --- | --- | --- | --- | --- | --- | --- | --- | --- | --- | --- | --- | --- | --- | --- | --- | --- | --- | --- | --- | --- | --- | --- |
|  |  | **MMU-5/9/14*** | | **MMU-5/7/19*** | **MMU-5/7/10***** | | | **MMU - 5/10*** |  | **MMU-3/18*** | **MMU-8/13*** | | | **MMU-6/12*** | | | |  |  | |  | | |
| **Famíly Cricetidae** |  |  | |  |  | | |  |  |  |  | | |  | | | |  |  | |  | | |
| **Subfamíly Sigmodontinae** |  |  | |  |  | | |  |  |  |  | | |  | | | |  |  | |  | | |
| **Tribe Akodontini** |  |  | |  |  | | |  |  |  |  | | |  | | | |  |  | |  | | |
| *Akodon cursor* | 15 |  | | MMU-5/7/19 |  | | |  |  | MMU-3/18 | MMU-8/13 | | | | MMU-6/12 | | | | |  | |  | 21 |
| *Akodon montensis* | 24 |  | | MMU-7/19 |  | | |  |  | MMU-3/18 | MMU-8/13 | | | | MMU-6/12 | | | | |  | |  | 21 |
| *Akodon paranaensis* | 44 |  | | MMU-7/19 |  | | |  |  | MMU-3/18 | MMU-8/13 | | | | MMU-6prox/12ent | | | | |  | |  | 21 |
| *Akodon serrensis* | 46 |  | | MMU-7/19 |  | | |  |  | MMU-3/18 | MMU-8/13 | | | | MMU-6prox/12ent | | | | |  | |  | 21 |
| *Necromys lasiurus* | 34 | MMU-5/9/14 | | MMU-5/7/19 |  | | | MMU - 5/10 |  | MMU-3/18 | MMU-8/13 | | | | MMU-6prox/12ent | | | | |  | |  | 22 |
| *Thaptomys nigrita* | 52 |  | | MMU-7/19 |  | | |  |  |  | MMU-8/13 | | | |  | | | | |  | |  | 22 |
| **Tribe Oryzomyini** |  |  | |  |  | | |  |  |  |  | | | |  | | | | |  | |  |  |
| *Oligoryzomys flavescens* | 64 |  | | MMU-7/19 |  | | |  |  |  | MMU-8/13 | | | |  | | | | |  | |  | 21 |
|  |  |  | |  |  | | |  |  |  |  | | |  | | | |  |  | |  | | |
| **Subfamily Cricetinae** |  |  | |  |  | | |  |  |  |  | | |  | | | |  |  | |  | | |
| *Cricetulus griseus* | 22 | MMU-5/9 | | MMU-7/19 |  | | |  |  |  |  | | |  | | | |  |  | | 40, 45 | | |
| *Cricetus cricetus* | 22 | MMU-5/9 | | MMU-7/19 |  | | |  |  |  |  | | |  | | | |  |  | | 46 | | |
| *Mesocricetus auratus* | 44 |  | | MMU-7/19 |  | | |  |  |  |  | | |  | | | |  |  | | 40 | | |
|  |  |  | |  |  | | |  |  |  |  | | |  | | | |  |  | |  | | |
| **Subfamily Arvicolinae** |  |  | |  |  | | |  |  |  |  | | |  | | | |  |  | |  | | |
| *Ellobius lutescens* | 17 |  | | MMU-7/19 | MMU-7/5/10 | | |  |  |  |  | | | MMU-6med+dist/12prox | | | | | |  | |  | 41 |
| *Ellobius talpinus* | 54 |  | | MMU-7/19 | MMU-7/5/10 | | |  |  |  |  | | | MMU-6med+dist/12prox | | | | | |  | |  | 41 |
| *Microtus agrestis* | 50 |  | | MMU-7/19 | MMU-7/5/10 | | |  |  |  |  | | | MMU-6med+dist/12prox | | | | | |  | |  | 41 |
| *Microtus oeconomus* | 30 |  | | MMU-7/19 | MMU-7/5/10 | | |  |  |  |  | | | MMU-6med+dist/12prox | | | | | |  | |  | 42 |
|  |  |  | |  |  | | |  |  |  |  | | |  | | | |  |  | |  | | |
| **Subfamily Neotomyinae** |  |  | |  |  | | |  |  |  |  | | |  | | | |  |  | |  | | |
| *Peromyscus maniculatus* | 48 |  | | MMU-7/19 |  | | |  |  |  |  | | |  | | | |  |  | | 36 | | |
| *Peromyscus eremicus* | 48 |  | | MMU-7/19 |  | | |  |  |  |  | | |  | | | |  |  | | 46 | | |
|  |  |  | |  |  | | |  |  |  |  | | |  | | | |  |  | |  | | |
| **Family Muridae** |  |  | |  |  | | |  |  |  |  | | |  | | | |  |  | |  | | |
| **Subfamily Murinae** |  |  | |  |  | | |  |  |  |  | | |  | | | |  |  | |  | | |
| *Apodemus agrarius* | 48+Bs |  | | MMU-7/19 |  | | |  |  |  |  | | |  | | | |  |  | | 35 | | |
| *Apodemus argenteus* | 46+Bs |  | | MMU-7/19 |  | | |  |  |  |  | | |  | | | |  |  | | 35 | | |
| *Apodemus gurkha* | 48 |  | | MMU-7/19 |  | | |  |  |  |  | | |  | | | |  |  | | 35 | | |
| *Apodemus peninsulae* | 48+Bs |  | | MMU-7/19 |  | | |  |  |  |  | | |  | | | |  |  | | 35 | | |
| *Apodemus semotus* | 48 |  | | MMU-7/19 |  | | |  |  |  |  | | |  | | | |  |  | | 35 | | |
| *Apodemus speciosus* | 46 or 48 |  | | MMU-7/19 |  | | |  |  |  |  | | |  | | | |  |  | | 35 | | |
| *Apodemus sylvaticus* | 48+Bs |  | | MMU-7/19 |  | | |  |  |  |  | | |  | | | |  |  | | 35 | | |
| *Coelomys pahari (Mus)* | 48 |  | | MMU-7/19 |  | | |  |  |  |  | | |  | | | |  |  | | 44 | | |
| *Micromys minutus* | 68 |  | | MMU-7/19 |  | | |  |  |  |  | | |  | | | |  |  | | 38 | | |
| *Millardia metalda* | 50 |  | | MMU-7/19 |  | | |  |  |  |  | | |  | | | |  |  | | 38 | | |
| *Mus platythrix* | 26 |  | | MMU-7/19 |  | | |  |  |  |  | | |  | | | |  |  | | 34 | | |
| *Nannomys mattheyi* | 36 | MMU-5/9 | | MMU-7/19 |  | | |  |  |  |  | | |  | | | |  |  | | 44 | | |
| *Praomys tullbergi* | 34 |  | | MMU-7/19 |  | | |  |  |  |  | | |  | | | |  |  | | 32 | | |
| *Rattus norvegicus* | 42 |  | MMU-7/19 | | |  |  | |  |  | |  |  | | |  |  | 43, 45 | | | | | |
| *Rattus rattus* | 38 |  | MMU-7/19 | | |  |  | |  |  | |  |  | | |  |  | 31 | | | | | |
| *Rhabdomys pumilio* | 46 |  | MMU-7/19 | | |  |  | |  |  | |  |  | | |  |  | 39 | | | | | |
| *Tokudaia osimensis* | 25 |  | MMU-7/19 | | |  |  | |  |  | |  |  | | |  |  | 35 | | | | | |
| *Tokudaia tokunoshimensis* | 45 |  | MMU-7/19 | | |  |  | |  |  | |  |  | | |  |  | 35 | | | | | |
|  |  |  |  | | |  |  | |  |  | |  |  | | |  |  |  | | | | | |
| **Subfamily Deomyinae** |  |  |  | | |  |  | |  |  | |  |  | | |  |  |  | | | | | |
| *Acomys dimidiatus* | 38 | MMU-5/9 | MMU-7/19 | | |  |  | |  |  | |  |  | | |  |  | 38 | | | | | |
|  |  |  |  | | |  |  | |  |  | |  |  | | |  |  |  | | | | | |
| **Subfamily Otomyinae** |  |  |  | | |  |  | |  |  | |  |  | | |  |  |  | | | | | |
| *Otomys irroratus* | 29 |  | MMU-7/19 | | |  |  | |  |  | |  |  | | |  |  | 33 | | | | | |
|  |  |  |  | | |  |  | |  |  | |  |  | | |  |  |  | | | | | |
| **Ancestral Muroidea Karyotype** |  |  |  | | |  |  | |  |  | |  |  | | |  |  |  | | | | | |
| AMK | 48/50 or 52 |  | MMU-7/19 | | |  |  | |  |  | |  |  | | |  |  | 24, 32, 40, 46, 52 | | | | | |

* **Shared blocks between *Necromys lasiurus* and other species analyzed in this study.**

** **Reference following manuscript the present study:** **21** - Hass et al. 2008; **22** - Hass et al. 2011; **24** – Romanenko et al. 2012; **31** - Cavagna et al. 2002; **32** - Chaves et al. 2012; **33** - Engelbrecht et al. 2006; **34** - Matsubara et al. 2003; **35** - Matsubara et al. 2004; **36** - Mlynarski et al. 2008; **37** - Nakamura et al. 2007a; **38** – Nakamura et al. 2007b; **39** - Rambau & Robinson, 2003; **40** - Romanenko et al. 2006; **41** - Romanenko et al. 2007a; **42** - Sitnikova et al. 2007; **43** - Stanyon et al. 1999; **44** - Veyrunes et al. 2006; **45** - Yang et al. 2000; **46** – Vieira-da-Silva et al. 2015; **52** - Romanenko et al. 2007.

*****Secondary characters obtained during analyses.**

**References**

**21** - Hass I, Sbalqueiro IJ, Müller S. Chromosomal phylogeny of four Akodontini species (Rodentia, Cricetidae) from Southern Brazil established by Zoo-FISH using *Mus musculus* (Muridae) painting probes. Chromosome Res. 2008; 16(1): 75-88 doi: 10.1007/s10577-007-1211-5 PMID: 18293106

**22** - Hass I, Müller S, Artoni RF, Sbalqueiro IJ. Comparative Chromosome Maps of Neotropical Rodents *Necromys lasiurus* and *Thaptomys nigrita* (Cricetidae) Established by ZOO-FISH. Cytogenet Genome Res. 2011; 135(1): 42–50 doi: 10.1159/000330259 PMID: 21846965

**24** - Romanenko SA, Perelman PL, Trifonov VA, Graphodatsky AS. Chromosomal evolution in Rodentia. Heredity. 2012; 108:4–16. doi: 10.1038/hdy.2011.110 PMID:22086076

**31** - Cavagna P, Stone G, Stanyon R. Black rat *(Rattus rattus)* genomic variability characterized by chromosome painting. Mamm. Genome. 2002, 13:157-163. PMID: 11919687

**32** - Chaves R, Louzada S, Meles S, Wienberg J, Adega F. Praomys tullbergi (Muridae, Rodentia) genome architecture decoded by comparative chromosome painting with Mus and Rattus.Chromosome Res. 2012, 20:673-683. doi: 10.1007/s10577-012-9304-1 PMID: 22847644

**33** - Engelbrecht A, Dobigny G, Robinson TJ. Further insights into the ancestral murine karyotype: the contribution of the Otomys – Mus comparison using chromosome painting. Cytogenet Genome Res. 2006, 112:126–130. PMID: 16276101

**34** - Matsubara K, Nishida-Umehara C, Kuroiwa A, Tsuchiya K, Matsuda Y. Identification of chromosome rearrangements between the laboratory mouse (Mus musculus) and the Indian spiny mouse (Mus platythrix) by comparative FISH analysis. Chromosome Res. 2003, 11: 57-64. PMID: 12675306**32** - Matsubara K., Nishida-Umehara C., Tsuchiya K., Nukaya D. and Matsuda Y. 2004 Karyotypic evolution of Apodemus (Muridae, Rodentia) inferred from comparative FISH analyses. Chromosome Research, 12: 383–395.

**35.** Matsubara K, Nishida-Umehara C, Tsuchiya K, Nukaya D, Matsuda Y. Karyotypic evolution of Apodemus (Muridae, Rodentia) inferred from comparative FISH analyses. Chromosome Res. 2004, 12: 383–395. PMID: 15241017

**36** - Mlynarski EE, Obergfell CJ, O’Neill MJ, O’Neill RJ. Divergent patterns of breakpoint reuse in Muroid rodents. Mamm Genome. 2010, 21:77–87. doi: 10.1007/s00335-009-9242-1 PMID: 20033182

**37** - Nakamura T, Kuroiwa A, Nishida-Umehara C, Matsubara K, Yamada F, Matsuda Y. Comparative chromosome painting map between two Ryukyu spiny rat species, Tokudaia osimensis and Tokudaia tokunoshimensis (Muridae, Rodentia). Chromosome Res. 2007a, 15:799–806. PMID: 17874214

**38** - Nakamura T, Matsubara K, Yasuda SP, Tsuchiya K, Matsuda Y. Chromosome homology between mouse and three Muridae species, Millardia meltada, Acomys dimidiatus and Micromys minutus, and conserved chromosome segments in murid karyotypes. Chromosome Res. 2007b, 15:1023–1032. PMID: 18095177

**39** - Rambau RV, Robinson TJ. Chromosome painting in the African four-striped mouse Rhabdomys pumilio: Detection of possible murid specific contiguous segment combinations. Chromosome Res. 2003, 11: 91-98. PMID: 12733636

**40** - Romanenko AS, Perelman PL, Serdukova NA, Trifonov VA, Biltueva LS, Wang J, et al. Reciprocal chromosome painting between three laboratory rodent species. Mamm Genome. 2006; 17(12): 1183-1192. PMID: 17143584

**41** - Romanenko SA, Sitnikova NA, Serdukova NA, Perelman PL, Rubtsova NV, Bakloushinskaya IY, et al. Chromosomal evolution of Arvicolinae (Cricetidae, Rodentia). II. The genome homology of two mole voles (genus Ellobius), the field vole and golden hamster revealed by comparative chromosome painting. Chromosome Res. 2007a, 15:891–897. PMID: 17924201

**42** - Sitnikova NA, Romanenko SA, O’Brien PCM, Perelman PL, Fu B, Rubtsova NV, et al. Chromosomal evolution of Arvicolinae (Cricetidae, Rodentia). I. The genome homology of tundra vole, field vole, mouse and golden hamster revealed by comparative chromosome painting. Chromosome Res. 2007; 15(4): 447-456 PMID: 17497247

**43** - Stanyon R, Yang F, Cavagna P, O’Brien PCM, Bagga M, Ferguson-Smith MA et al. Reciprocal chromosome painting shows that genomic rearrangement between rat and mouse proceeds ten times faster than between humans and cats. Cytogenet Cell Genet. 1999; 84(3-4): 150–155 PMID: 10393417

**44** - Veyrunes F, Dobigny G, Yang F, O'Brien PCM, Catalan J, Robinson TJ, et al. Phylogenomics of the genus**Mus**(Rodentia; Muridae): extensive genome repatterning is not restricted to the house mouse. Proc Biol Sci. 2006; 273(1604): 2925–2934. doi: 0.1098/rspb.2006.3670 PMID: 17015352

**45** - Yang F, O'Brien PCM, Ferguson-Smith MA. Comparative chromosome map of the laboratory mouse and Chinese hamster defined by reciprocal chromosome painting. Chromosome Res. 2000; 8: 219-227. PMID: 10841049

**46 -** Vieira-da-Silva A, Louzada S, Adega F, Chaves R. A High-Resolution Comparative Chromosome Map of *Cricetus cricetus* and *Peromyscus eremicus* Reveals the Involvement of Constitutive Heterochromatin in Breakpoint Regions. Cytogenet Genome Res. 2015; 145:59–67. doi: 10.1159/000381840 PMID:25999143

**52** - Romanenko SA, Volobouev VT, Perelman PL, Lebedev VS, Serdukova NA, Trifonov VA, et al. Karyotype evolution and phylogenetic relationships of hamsters (Cricetidae, Muroidea, Rodentia) inferred from chromosomal painting and banding comparison. Chromosome Res. 2007b; 15(3): 283-297 PMID: 17333534
